# Quantitative Comparison of HTLV-1 and HIV-1 Cell-to-Cell Infection with New Replication Dependent Vectors

**DOI:** 10.1371/journal.ppat.1000788

**Published:** 2010-02-26

**Authors:** Dmitriy Mazurov, Anna Ilinskaya, Gisela Heidecker, Patricia Lloyd, David Derse

**Affiliations:** HIV Drug Resistance Program, National Cancer Institute and SAIC-Frederick, NCI-Frederick, Frederick, Maryland, United States of America; Northwestern University, United States of America

## Abstract

We have developed an efficient method to quantify cell-to-cell infection with single-cycle, replication dependent reporter vectors. This system was used to examine the mechanisms of infection with HTLV-1 and HIV-1 vectors in lymphocyte cell lines. Effector cells transfected with reporter vector, packaging vector, and Env expression plasmid produced virus-like particles that transduced reporter gene activity into cocultured target cells with zero background. Reporter gene expression was detected exclusively in target cells and required an Env-expression plasmid and a viral packaging vector, which provided essential structural and enzymatic proteins for virus replication. Cell-cell fusion did not contribute to infection, as reporter protein was rarely detected in syncytia. Coculture of transfected Jurkat T cells and target Raji/CD4 B cells enhanced HIV-1 infection two fold and HTLV-1 infection ten thousand fold in comparison with cell-free infection of Raji/CD4 cells. Agents that interfere with actin and tubulin polymerization strongly inhibited HTLV-1 and modestly decreased HIV-1 cell-to-cell infection, an indication that cytoskeletal remodeling was more important for HTLV-1 transmission. Time course studies showed that HTLV-1 transmission occurred very rapidly after cell mixing, whereas slower kinetics of HIV-1 coculture infection implies a different mechanism of infectious transmission. HTLV-1 Tax was demonstrated to play an important role in altering cell-cell interactions that enhance virus infection and replication. Interestingly, superantigen-induced synapses between Jurkat cells and Raji/CD4 cells did not enhance infection for either HTLV-1 or HIV-1. In general, the dependence on cell-to-cell infection was determined by the virus, the effector and target cell types, and by the nature of the cell-cell interaction.

## Author Summary

Cell-free virus particles released from infected cells can be transmitted to target cells by diffusion or may be conveyed directly to target cells via specific intercellular contacts; the latter is referred to as cell-to-cell infection. Microscopic imaging has shown how viral proteins and virus particles move within and between cells, accumulating at sites of cell-cell contact. While we suspect that these images represent virus infection, it has been difficult to accurately quantify virus replication and provirus formation in most cell-to-cell infection experiments. Retroviral vectors that encode reporter proteins have been invaluable tools for analyzing retrovirus replication and restriction, but they have had limited utility in cell-to-cell infection studies due to high background noise resulting from reporter expression in the producer cells. We report the construction and characterization of retroviral vectors that express reporter protein exclusively in target cells and only after completing a full replication cycle. We have validated this approach and have begun to analyze cell and virus determinants for cell-to-cell infection with vectors for two human retroviruses that infect T cells. We show that the mechanism of transmission and ensuing virus replication depend on the particular virus, the effector and target cell types, and on the specific type of cell-cell interaction.

## Introduction

Retroviruses can infect cells as cell-free particles or by cell-to-cell transmission [1],[2],[3],[4],[5]. In the latter route of infection, specific cell-cell contacts may strongly enhance virus infection by triggering the reorganization of cytoskeletal and cell-surface protein networks to focus virus release toward clustered receptors on an apposed target cell [2],[6],[7],[8],[9],[10],[11],[12]. Cell-to-cell infection would require steps in the virus infectious cycle to be integrated with events in the cell-cell adhesion process; hence, the mechanism of cell-to-cell transmission would depend on specific interactions between cell and virus proteins. HTLV-1 is a highly cell-associated virus that is most likely disseminated by cell-to-cell transmission *in vivo* [13]. Microscopic image analysis of HTLV-1-infected lymphocytes in close contact with uninfected cells *in vitro* showed aggregation and transfer of virus components at a “virological synapse” (VS) [14],[15]; whether the transfer of viral proteins between cells was accompanied by provirus formation is still unknown. On the other hand, HIV-1 infection has been studied intensively, and *in vitro* systems have been used to examine cell-to-cell transmission of virus from infected T-cells or infected macrophages to uninfected T-cells and epithelial cells [2],[6],[7],[8],[9],[10],[11],[12],[16],[17],[18], as well as the special situation where HIV-1 particles are collected on dendritic cells and transmitted to T-cells via an “infectious synapse” [19],[20],[21]. MLV has been shown to move between cells along filipodial bridges that connect infected and uninfected cells not in immediate proximity [22]. More recently, a variety of cell-cell communicative structures such as nanotubes, mono- and polysynapses, have been demonstrated to serve as platforms for directed HIV particle egress, transfer and endocytosis by target cells [23]. In sum, our knowledge of retrovirus biology combined with *in vitro* experimental data suggests that cell-to-cell transmission is an important mechanism of virus spread *in vivo*.

Much of what we know about cell-to-cell infection is inferred from microscopic image analysis; fluorescent microscopy shows viral proteins mobilized to cell-cell contact sites and electron micrographs show virus particles localized between interacting cells [2],[4],[10],[14],[16],[24]. Direct evidence for virus replication in the context of cell-to-cell transmission has been reported for HIV-1 by measuring reverse transcription products in infected target cells or by FACS analysis of HIV-1 protein expression in fluorescently labeled target cells [6],[7],[11],[12],[25] or by long-term video microscopy observation of HIV Gag-iGFP replication [26]. Retroviral vectors, which have greatly facilitated studies of cell-free infection, are not well suited for examining cell-to-cell infection. One problem is that reporter gene expression in the producer cells generates a strong signal, and because these cells cannot be removed entirely from newly infected target cells, they obscure infection events. Clearly, a simple method to quantify cell-to-cell infection would provide a needed functional complement to image analysis, and help to define mechanisms of cell-to-cell infection.

We have solved these technical problems by constructing HIV-1 and HTLV-1 vectors that consist of a virus packaging plasmid, an Env-expression plasmid, and a replication dependent reporter vector. The design of the new reporter vectors is based on a concept described initially by Heidmann et al. [27], which was later adapted to study retrotransposition of endogenous retroviruses [28], mammalian LINE1 elements [29], and yeast TY1 elements [30]. We demonstrate here that the new HIV-1 and HTLV-1 reporter vectors are ideally suited for studying cell-to-cell infection, as reporter protein expression is confined exclusively to the infected target cell. By using eYFP based transfer vectors, we track infected cells that are not multinucleated and thereby rule out cell fusion as a mechanism of viral transmission. Quantifying the transduction of the new luciferase based vectors in target cells, we show the absolute dependence of HTLV-1 transmission on cell-cell contact, cytoskeleton remodeling and Tax protein expression, while HIV infection is enhanced twofold in our cell coculture settings and has characteristics both of cell-free and cell-to-cell modes of transmission. Induction of an immunological synapse (IS) between Jurkat effector cells and Raji/CD4 target cells does not increase infection with either HIV-1 or HTLV-1 VLPs suggesting that cell-to-cell infection requires the formation of specialized VS.

## Results

### Quantitation of HTLV-1 and HIV-1 infection in cocultures of VLP-producer cells and target cells

**Figure 1:**
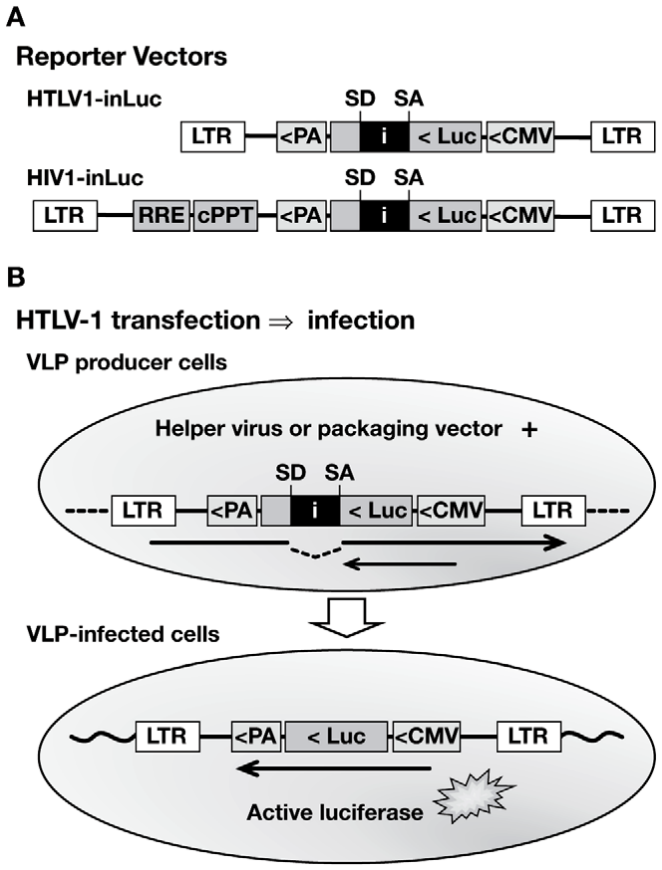
Specialized HTLV-1 and HIV-1 reporter vectors to measure single-cycle replication in coculture infection. (**A**) Replication-dependent HTLV-1 and HIV-1 transfer vectors contain an antisense-oriented expression cassette, which consists of a CMV promoter, firefly luciferase (luc) gene, and TK polyA signal (PA). The luc gene is interrupted by an intron (*i*), which is oriented in the sense direction, indicated by splice donor (SD) and splice acceptor (SA) sites. The HIV1-inLuc vector contains a Rev responsive element (RRE) and central polypurine tract (cPPT). Analogous vectors were constructed that contain a yellow fluorescent protein gene with intron (inYFP). (**B**) Translation of the reporter protein mRNA (small arrow) in transfected cells is blocked by the intron. The vector mRNA (large arrow) is spliced and packaged into VLPs; after infection and replication, a provirus is formed that lacks the intron and is capable of reporter gene expression.

**Figure 2:**
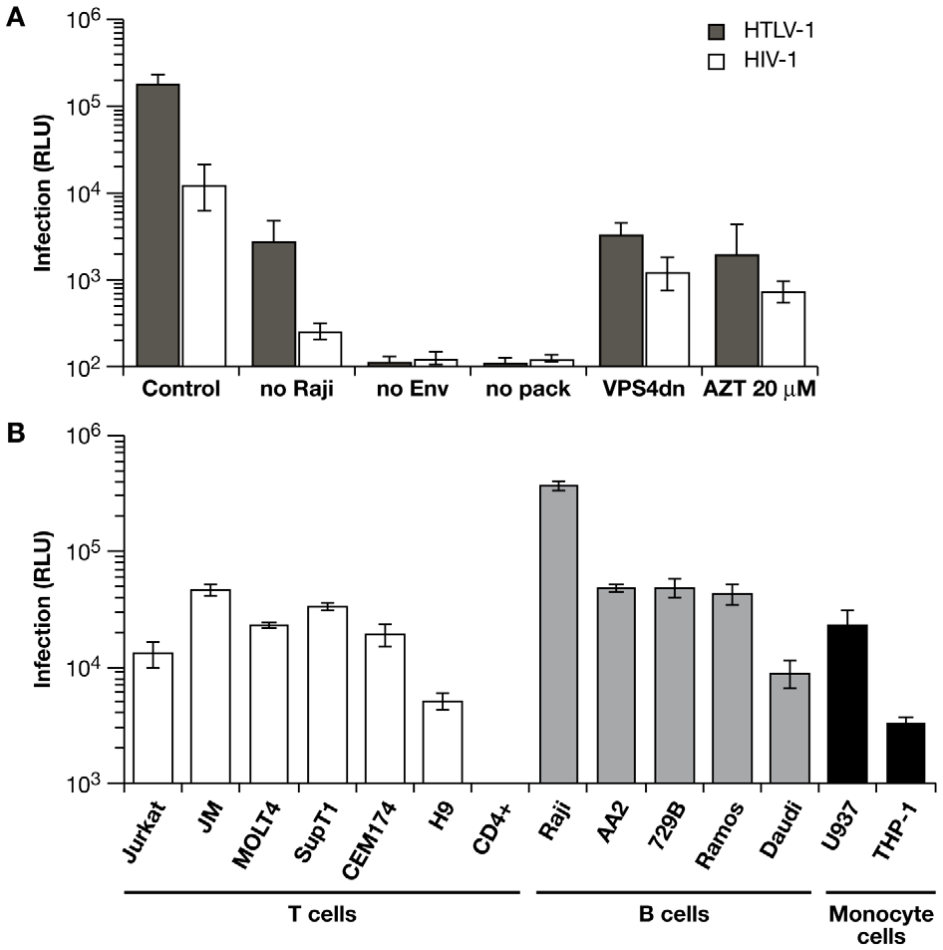
Characteristics of the replication dependent vectors in coculture infections. (**A**) HTLV1-inLuc and HIV1-inLuc vectors were transfected into Jurkat cells with respective packaging vectors and Env expression plasmids; transfected Jurkat cells were combined with an equal number of Raji/CD4 cells 24 h later. Infection is expressed as relative light units (RLU) of luciferase activity, measured 48 h after cell mixing. Coculture of Raji/CD4 cells with Jurkat cells transfected with wild type vectors is designated as control. The level of infection between Jurkat cells was determined in cultures without Raji/CD4 cells (no Raji). In other cocultures, Jurkat cells transfected with reporter vectors but without Env-expression plasmid (no Env) or without packaging plasmid (no pack) produced no Luc activity above background. Cotransfection of the viral vectors with a dominant-negative VPS4A plasmid (VPSdn), which inhibits virus budding, or addition of the reverse transcriptase inhibitor azidotimidine (AZT 20 µm) inhibited Luc transduction. (**B**) Jurkat cells were transfected with HTLV-1 packaging plasmid and HTLV1-inLuc reporter vector and then incubated with an equal number of the indicated cells. Luc activity in the target cells was assayed 48 h after cell mixing. Luc activity was not detected in activated human CD4^+^ T cells from different donors in repeated experiments. Graphs represent the mean of at least three independent experiments with error bars indicating standard deviations.

In order to take advantage of the sensitivity and versatility of retroviral vectors for studying virus replication, and to overcome difficulties encountered with standard reporter vectors in coculture infection experiments, we constructed reporter vectors similar to those that have been used to study retrotransposition of various mobile genetic elements [28],[29],[30]. The new HTLV-1 and HIV-1 reporter vectors contain a reporter gene cassette in antisense orientation relative to the virus; the reporter gene is interrupted by an intron, which is oriented in the sense direction (Fig. 1A). The intron, which can be spliced only from the vector mRNA, prevents expression of the reporter gene in transfected effector cells (Fig. 1B). Virus-like particles (VLPs) are produced after transfection of effector cells with reporter vector and virus packaging vector. Infection of target cells with VLPs that contain the spliced reporter vector RNA will generate a provirus that is now capable of expressing the reporter protein (Figure 1B). HTLV-1 and HIV-1 reporter vectors were made that encode either luciferase (inLuc) or yellow fluorescent protein (inYFP) genes. When transfected alone into 293T cells (not shown) or Jurkat cells (Fig. 2A), the inLuc vectors did not express detectable levels of luciferase activity. VLPs produced from cells transfected with either HTLV-1 or HIV-1 vectors contain reporter vector mRNA of which approximately 35% has no intron (data not shown).

To validate these vectors and to examine the mechanisms of retrovirus transmission between cells, we developed a model coculture system with Jurkat T cells as the VLP producers. Jurkat cells are well characterized, have high transfection efficiency, and mimic virus-producing T cells. For reasons described below, Raji/CD4 cells were used as targets [31]. Jurkat cells were transfected with viral vectors and incubated for 24 h before adding an equal number of Raji/CD4 target cells and infections were quantitated by luciferase assay 48 h after the start of coculture. Transduction of Luc activity was higher with HTLV-1 vectors compared to HIV-1 vectors (Fig. 2A); this is notable because cell-free infection with HIV-1 and HTLV-1 VLPs shows the opposite (see below, section ‘Differences in HTLV-1 and HIV-1 transmission in the coculture infections’). Transduction of Luc activity was 20-fold to 50-fold lower for both viruses when Raji/CD4 target cells were absent; i.e. when VLPs were transmitted between Jurkat cells. In the absence of either an Env expression vector or a viral packaging plasmid, no reporter gene activity was detected (Fig. 2A). VLP infectivity required virus budding, as cotransfection of the viral vectors with a dominant negative VPS4A expression plasmid inhibited Luc transduction. Treatment of cells with azidothymidine (AZT) also inhibited VLP infection, indicating the requirement for reverse transcriptase. In addition, mutations of Gag late domains or of viral protease, reverse transcriptase, and integrase genes in the viral packaging plasmids abolished Luc transduction (data not shown). These results indicated that the HIV-1 and HTLV-1 vectors transduced target cells in coculture with no background from the effector cells. Furthermore, cell-cell fusion did not contribute to infection or reporter gene expression (see Fig. S1 and Protocol S1).

To identify suitable target cells for coculture infection experiments, we examined Luc transduction of established cell lines and primary activated CD4^+^ T cells by coculture with Jurkat cells transfected with HTLV-1 vectors (Fig. 2B). T-cell lines, such as Jurkat, JM, MOLT4, SupT1, and CEM174, yielded similar levels of Luc activity 48 h after the start of coculture with Jurkat effector cells; H9 cells gave somewhat lower levels of Luc activity compared to the other T-cell lines. Unexpectedly, and in repeated attempts with cells from various donors, we were unable to detect Luc activity after coculture of Jurkat effector cells with activated human CD4^+^ T cells. In parallel experiments, the same CD4^+^ T cells were efficiently transduced with cell-free HIV-1 VLPs. It is presently unclear whether infection, provirus formation, or subsequent reporter gene expression are inhibited during the 48 h of coculture of activated CD4^+^ T cells with Jurkat effector cells; we are currently examining the reasons for this inhibitory effect. It was not surprising that HTLV-1 vectors transduced B-cell and monocyte cell lines, as it is well known that HTLV-1 infects a wide variety of cell types *in vitro* via Env interactions with ubiquitously expressed receptor(s), GLUT-1, NP1, and heparan sulfate proteoglycans [32],[33],[34]. In general, HTLV-1 transduction of B-cell lines appeared to be higher than the T-cell lines, with Raji/CD4 cells giving the highest level of Luc activity of any cell line tested. It is not clear yet whether the high level of Luc activity detected in Raji/CD4 cells is due to specific interactions with Jurkat cells that enhance virus transmission and replication, or that Raji/CD4 cells support higher levels of reporter gene expression. In addition to the high levels Luc transduction, we chose to use Raji/CD4 cells in the following experiments for several reasons. First, B-cells are natural targets for HTLV-1 infection *in vivo* and *in vitro* [35],[36],[37],[38],[39]. Furthermore, B-cells were shown to form conjugates (virological synapses) with HTLV-1-infected T cells in PBMC cultures from HTLV-1-infected individuals [14]. Second, while we recognize that B-cells are not natural targets for HIV-1 infection, Raji/CD4 cells can be infected by both HTLV-1 and HIV-1 vectors for comparative analyses. Finally, Jurkat and Raji cells have been used previously to study immunological synapse formation [40],[41], and in experiments described below, this allowed us to determine whether forcing cells together via a superantigen-induced synapse would enhance or inhibit virus infection.

### Role of the cytoskeleton in HTLV-1 and HIV-1 coculture infection

**Figure 3:**
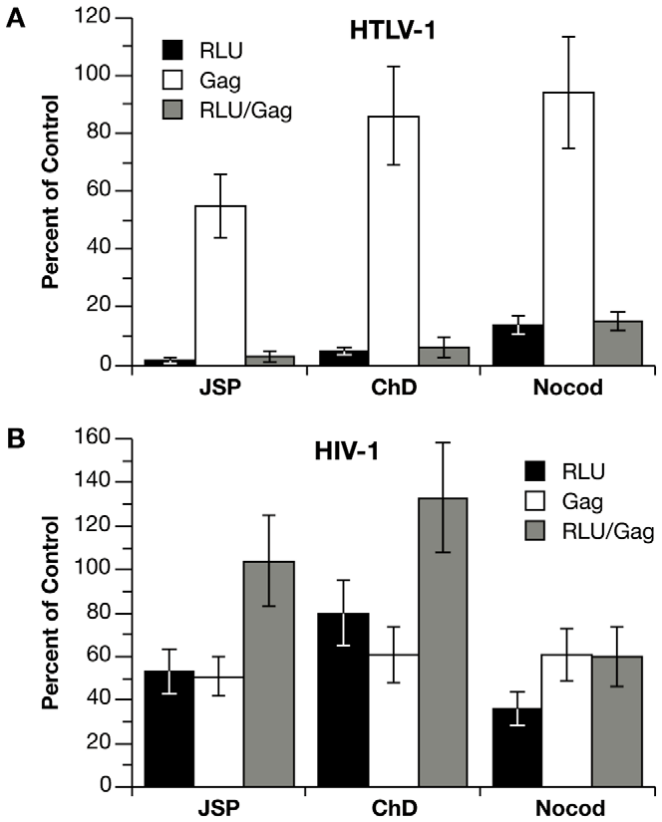
Effects of cytoskeleton disrupting agents on coculture infection with HTLV-1 and HIV-1 vectors. (**A**) Jurkat cells were transfected with HTLV-1 vectors or (**B**) HIV-1 vectors (plus HTLV-1 Tax expression plasmid) and then mixed with an equal number of Raji/CD4 cells. Cocultures were left untreated or treated for the duration of the coculture with 100 nM jasplakinolide (Jsp), 5 µM cytochalasin D (ChD), or 5 µM nocodozole (nocod). Values for infection (RLU of Luc activity) and Gag concentration in culture supernatants (Gag) are expressed as percent relative to untreated controls. For HTLV-1 vectors, Luc activity was 3.54×10^5^ RLU and Gag (p19) concentration was 1.18 ng per ml in the untreated control. For HIV-1 vectors, Luc activity was 2.83×10^5^ RLU and Gag (p24) concentration was 1.81 ng per ml in the untreated control. Graphs represent the mean of at least three independent experiments with error bars indicating standard deviations.

To examine the role of cytoskeletal remodeling on single-cycle infection with HTLV-1 and HIV-1 vectors in the Jurkat-Raji/CD4 system, cocultures were treated with cytochalasin D (ChD) or jasplakinolide (Jsp), which target actin polymerization/depolymerization, or with nocodazole, which inhibits tubulin polymerization. Because of the reversible effects of the inhibitors and the fact that infection is measured 48 h after the start of coculture, both effector and target cells were exposed to the drugs for the duration of the experiment (Fig. 3). We observed that there was no significant decrease in cell-free infection of inhibitor-treated Raji/CD4 target cells with HTLV-1 and HIV-1 VLPs (data not shown). To control for the effects of the inhibitors on VLP production in effector cells, we measured Gag protein in cell culture supernatants at the end of the coculture experiment by HTLV-1 p19 or HIV-1 p24 ELISA. Figure 3 shows infectivity (Luc activity), Gag concentration in the supernatant, and normalized infectivity (Luc activity divided by Gag level) relative to untreated controls. The actin inhibitors, Jsp and ChD, diminished VLP production by 45% and 14% respectively with HTLV-1 vectors (Fig. 3A) and by 49% and 39% with HIV-1 vectors (Fig. 3B). For HIV-1 vectors, the decrease in infection resulting from Jsp and ChD treatment could be accounted for by the decrease in VLP production, because the normalized infectivity was equal to or greater than the untreated control. In contrast, the inhibition of infection with HTLV-1 vectors was significantly greater than the decrease in VLP production, and the normalized infectivity was 3% and 5% of untreated controls (Fig. 3A). Nocodozole had only a modest effect on HTLV-1 VLP production (6% decrease) but had a significant inhibitory effect on coculture infection (85% decrease) (Fig. 3A). For HIV-1 vectors, nocodazole inhibited both VLP production and infection, the normalized infectivity (60% compared to untreated control) was not as severely affected as HTLV-1. Together, the single-cycle replication data indicate that coculture infections with HTLV-1 vectors were strongly dependant on cytoskeletal remodeling, whereas infections with HIV-1 vectors were much less so in this experimental setting. These results suggest that the cell-free component of HIV-1 infection in Jurkat-Raji/CD4 co-culture predominate, but do not exclude an important role for cell-to-cell infectious transmission of HIV with other effector-target cell combinations. In contrast, HTLV-1 infection appears to require cell-cell contact.

### Differences in HTLV-1 and HIV-1 transmission in the coculture infections

**Figure 4:**
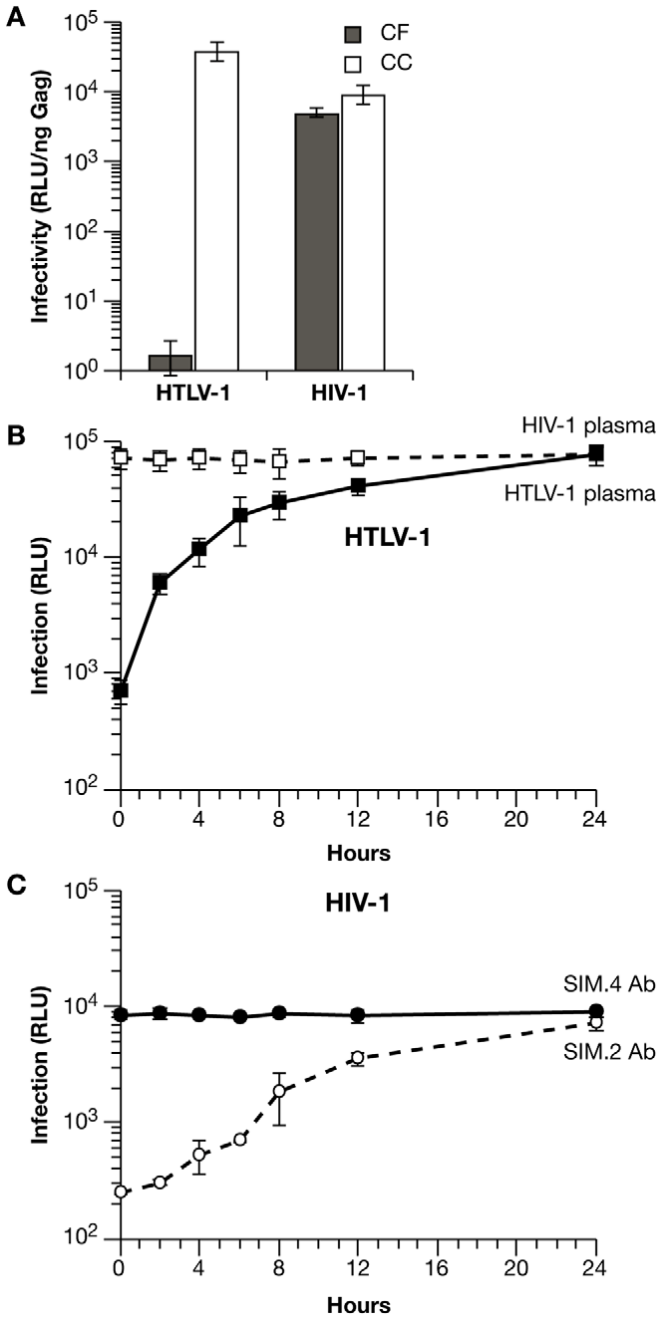
Differences in the mechanisms of transmission for HTLV-1 and HIV-1 in Jurkat-Raji/CD4 cocultures. (**A**) Comparison of cell-free (CF) and coculture (CC) infection. Raji/CD4 cells were infected with cell-free VLPs produced from transfected 293T cells or by coculture with transfected Jurkat cells. Luc activity is normalized relative to the amount of viral Gag protein in the supernatants. (**B**) The time course of HTLV-1 coculture infection was examined by blocking virus entry and infection with HTLV-1-infected human plasma (filled squares) at the indicated times after mixing transfected Jurkat cells and Raji/CD4 cells. Luc activity was measured 48 h after the start of coculture (0 h). For control infections, human plasma from HIV-1-infected patients was used (open squares). (**C**) Time course of HIV-1 coculture infection was examined by blocking virus entry and infection as in (B) except that anti-CD4 mAb SIM.2 (open circles) was used to block entry and non-blocking anti-CD4 mAb SIM.4 was used as control (filled circles). Luc activity was measured 48 h after the start of coculture (0 hr). These experiments were performed at least 3 times; error bars depict standard deviations.

We next compared the relative efficiency of cell-free versus cell-to-cell infection with HIV-1 and HTLV-1 vectors. Although VLP titers based on Gag ELISA are very similar for HTLV-1 and HIV-1 vectors, infectious titers are quite different [42],[43]. In order to obtain sufficiently high infectious titers of HTLV-1 VLPs for cell-free infection experiments, 293T cells were transfected with the same viral vectors and at a ratio identical to those used in transfection of Jurkat cells and luciferase transduction was normalized relative to the amount of Gag in the filtered supernatant (Fig. 4A). Infections in Jurkat-Raji/CD4 cocultures were carried out as before, where infection is normalized to the amount of Gag in the supernatant 48 h after mixing (Fig. 4A). For HTLV-1 vectors, infectivity was at least 4 logs higher in coculture infection compared with cell-free VLPs. In contrast, the infectivity of HIV-1 vectors was only 2-fold higher in cocultures compared with cell-free VLPs. These data are in agreement with the previous experiments and suggest a cell-to-cell mode of infection with HTLV-1 VLPs in Jurkat-Raji/CD4 cocultures, while cell free transmission constitutes a significant component of HIV-1 infection in this system.

In the Jurkat-Raji/CD4 coculture system, infection is measured 48 h after cell mixing, as this is the time required for optimal reporter gene expression. It would be desirable, however, to limit the period of VLP transmission to the first few hours after the start of coculture, as one would expect there to be significant differences in the levels of infection at these early times that are related to the mode of virus transmission. This can be accomplished by blocking virus entry at various times after cell mixing with either anti-Env neutralizing antibodies or antibodies that block virus receptors. For the purposes of this experiment, it does not matter which type of antibody is used, as it is important only to block infection after a defined period of virus transmission (Fig. S2). Time-course of transmission experiments, in which virus entry and infection were blocked with antiserum to HTLV-1 Env or antibody to the CD4 receptor, are shown in Fig. 4B and 4C. Anti-HTLV-1 human serum or anti-CD4 mAb were titrated to give greater than 95% inhibition of infection; control serum or mAb did not inhibit Luc transduction and neutralization of infection was specific for each virus. When antibodies were added at the same time that cells were combined (0 h), Luc transduction was inhibited by almost 2 orders of magnitude. For HTLV-1, levels of infection rose rapidly, increasing by about 10-fold during the first 4 h, then began to plateau after 6 h (Fig. 4B). In contrast, HIV-1 infection increased only 2-fold by 4 h after the start of coculture (Fig. 4C). These results indicate that the mechanisms of transmission of HTLV-1 and HIV-1 differ in this experimental system (see discussion below); for HTLV-1 VLPs, cell-to-cell spread appears to be the dominant mode of infection.

### Effects of HTLV-1 Tax on the mechanism of VLP transmission in Jurkat-Raji/CD4 cocultures

**Figure 5:**
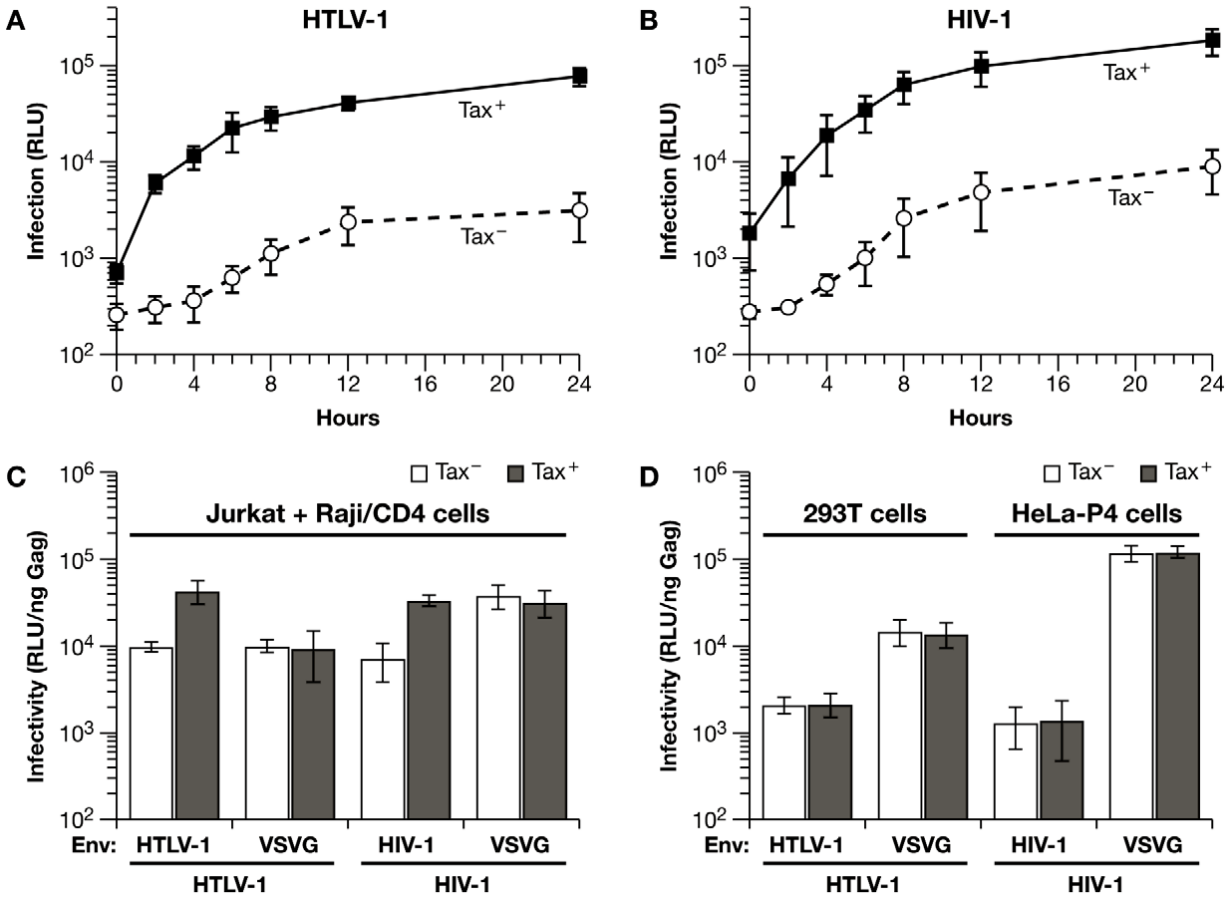
HTLV-1 Tax expression in Jurkat cells enhances coculture infection of Raji/CD4 cells with HIV-1 and HTLV-1 vectors. (**A**) Time course coculture infection experiments were performed as in figure 4B except that Jurkat cells were transfected with either wt (Tax+) or mutated (Tax^−^) HTLV-1 packaging vectors. (**B**) A coculture infection time course experiment was performed as in figure 4C except that Jurkat cells were transfected with HIV-1 vectors with either a Tax-expression vector (Tax^+^) or empty vector (Tax^−^). (**C**) HIV-1 and HTLV-1 VLPs were pseudotyped with VSV-G or their own Env and examined in Jurkat-Raji/CD4 coculture infection system with or without Tax expression. Infectivity is expressed as Luc activity (RLU) relative to the amount of Gag protein in the culture medium at 48 h. (**D**) Human 293T cells or HeLa-P4 cells were transfected with the same plasmids as in panel C. The medium was replaced the next day and the infection was allowed to develop until 48 h after transfection. Infectivity is expressed relative to the concentration of Gag in the supernatant. All results represent the mean of at least three independent experiments with standard deviations represented by error bars.

HTLV-1 Tax interacts with a variety of cellular proteins to alter transcription, signal transduction, cell adhesion, and cytoskeletal remodeling [44]. To determine whether Tax has an impact on coculture infections, HTLV-1 packaging plasmids were used that contain either a wild type (Tax^+^) or a mutated tax gene (Tax^−^); for HIV-1 infections, cells were cotransfected with HIV-1 vectors plus a Tax expression plasmid (Tax^+^) or empty vector (Tax^−^). We first examined the effects of HTLV-1 Tax expression on the time course of VLP transduction where infection was blocked with neutralizing antisera at various times after the start of coculture (Fig. 5A and B). With wt HTLV-1 vectors, Luc transduction increased rapidly over the first several hours of coculture (Fig. 5A). With the Tax^−^ vector, the initial change in HTLV-1 infection was significantly diminished and the time course resembled HIV-1 infection (Fig. 5A). When Tax was co-expressed with HIV-1 vectors, the initial rate of infection was increased significantly, nearly reaching the levels obtained with wild type HTLV-1 vectors (Fig. 5B). These results indicate that HTLV-1 Tax is a major determinant of the difference observed in the mechanism of HTLV-1 and HIV-1 transmission here. This is consistent with known effects of Tax on the expression and activity of adhesion proteins and activation of signal transduction pathways that may cooperate to enhance virus infection and replication.

To determine whether Env plays a role in determining the mechanism of virus transmission in coculture infections, we examined pseudotyped VLPs (Fig. 5C). Unfortunately, HTLV-1 VLPs pseudotyped with HIV-1 Env were not infectious and HIV-1 VLPs pseudotyped with HTLV-1 Env had very poor infectivity (Fig. S3). These results may reflect differences in Gag and Env trafficking between HTLV-1 and HIV-1, as we have previously shown that HIV-1 and HTLV-1 Gag are targeted to different plasma membrane microdomains for virion assembly in Jurkat cells [40]. However, both HIV-1 and HTLV-1 VLPs can be pseudotyped with VSV-G protein. Expression of Tax in Jurkat cells boosted infection by about 5-fold for both HTLV-1 and HIV-1 VLPs in Jurkat-Raji/CD4 cocultures (Fig. 5C). However, the Tax-induced enhancement of infection was not seen when VLPs were pseudotyped with VSV-G protein, suggesting that Tax elicits specific cellular alterations that enhance infection only in combination with a particular type of Env. We also examined the effects of Tax expression in cocultures of transfected 293T cells or HeLa-P4 cells (Fig. 5D). In this one-step transfection/infection coculture system, transfected cells produce VLPs that infect neighboring cells. Neither HTLV-1 nor HIV-1 VLP transduction was enhanced by Tax in 293T cells or HeLa-P4 cells (Fig. 5D) or in cell-free infections (data not shown). Together, these data indicate that Tax-mediated enhancement of infection in cocultures is dependent on cell type and on Env. Perhaps, Tax is not only involved in the mobilization of Gag to the cell synapse, but also in establishing crosstalk between certain Env and adhesion molecules for their efficient membrane movement toward the site of cell-cell contact.

### A superantigen-induced immunological synapse does not enhance cell-to-cell infection

**Figure 6:**
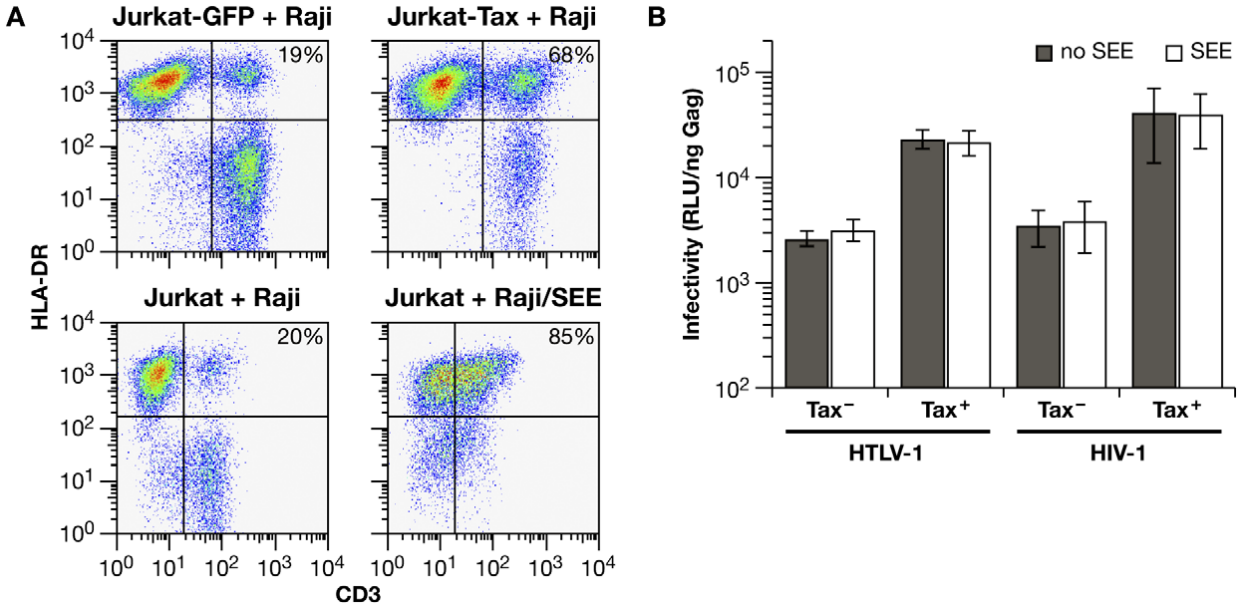
A superantigen-induced immunological synapse does not enhance infection with HIV-1 or HTLV-1 VLPs. (**A**) Conjugate formation between Jurkat cells and Raji/CD4 cells was quantified by flow cytometry. One hour after mixing, cells were fixed and stained with anti-CD3-FITC (Jurkat cells) and anti-HLA-DR-PE antibodies (Raji/CD4 cells); cell-cell conjugates are identified as double-positive signals. The basal level of conjugate formation was approximately 20% (left hand panels). In the upper panels, Jurkat cells were transduced with lentivirus vectors encoding GFP only (left) or Tax-IRES-GFP (right) 48 hrs before mixing with Raji/CD4 cells. In the lower panels, Raji/CD4 cells were untreated (left) or were pulsed with SEE (right) prior to mixing with Jurkat cells. Typical Dot Plots from one of three experiments are presented. (**B**) Effects of SEE-induced synapse formation on HIV-1 and HTLV-1 infection in Jurkat-Raji/CD4 cocultures. Luc activity is normalized relative to the amount of Gag in culture supernatants at the end of infection. The histograms represent the mean of four independent experiments.

It has been reported previously that HTLV-1 Tax induces homotypic aggregation in various T-cell lines [45]. To determine whether the ability of Tax to enhance infection in Jurkat-Raji/CD4 cocultures was simply due to cell-cell aggregation, we examined whether inducing a synapse between Jurkat cells and Raji/CD4 cells would affect cell-to-cell infection. Staphylococcal enterotoxin E (SEE) induces the formation of an immunological synapse (IS) by binding to both the TCR on Jurkat T cells and MHC-II on Raji B cells [46]. We used this system previously to show that HTLV-1 Gag traffics with tetraspanin-enriched plasma membrane microdomains to the IS [40]. Stable interaction between Jurkat cells and Raji/CD4 cells was assayed by flow cytometry (Fig. 6A). In the absence of Tax expression or SEE treatment, about 20% of Jurkat cells fractionated with Raji/CD4 cells (Fig. 6A, left hand panels). Transduction of Jurkat cells with a lentivirus vector expressing HTLV-1 Tax (approximately 85% transduction efficiency) before mixing with Raji/CD4 cells increased conjugate formation to 68%. When Raji/CD4 cells were pretreated with SEE and mixed with Jurkat cells, 85% of Jurkat cells were conjugated with Raji/CD4 cells (Fig. 6A, lower right hand panel). Coculture of transfected Jurkat cells with Raji/CD4 cells that had been pretreated with SEE revealed that induction of an IS did not enhance HIV-1 or HTLV-1 VLP infectivity, either in the presence or absence of Tax (Fig. 6B). These results are in striking contrast to the enhancement of infectivity by Tax, suggesting that Tax increases the expression or activity of specific adhesion molecules and signaling pathways necessary for efficient cell-to-cell infection.

## Discussion

Our aim was to develop retroviral vectors that would make it possible to directly quantify retroviral replication in cell-to-cell virus transmission experiments. Retroviral reporter vectors have greatly enhanced our understanding of the retrovirus infectious cycle, but their utility has been limited primarily to cell-free infection studies due to high levels of reporter gene expression in VLP producer cells. The new reporter vectors, referred to here as inLuc or inYFP vectors, rely on RNA splicing in the effector cell and provirus formation in the target cell to activate reporter gene expression. Considering the complex mechanisms that both HIV-1 and HTLV-1 have evolved to regulate mRNA splicing and transport, the system produces surprisingly clear results. There was no signal from the reporter vector alone in transfected cells and disrupting a viral structural or enzymatic function in the packaging vector abolished reporter protein expression. These vectors are ideally suited for studies of cell-to-cell infection in the coculture setting. In previous studies of virus transmission from a transfected or virus-infected effector cell to a target cell, infection was often inferred by measuring viral protein transfer between cells by microscopy, flow cytometry [11],[12], or by the formation of nascent reverse transcription products [6],[7]. Hubner W. at al. [26] beautifully demonstrated early and late stages of HIV cell-to-cell transmission and target cell infection using 3D video microscopy of replication competent fluorescent HIV clone. However, most of the above methods require virus-infected rather than transfected effector cells due to background problems associated with transfected plasmid DNA. It is therefore difficult to analyze viral mutants and pseudotyped virions. We believe that the vectors described here will help to mitigate some of these problems and enable future quantitative studies of cell-to-cell infection.

The new vectors were validated in coculture infections with transfected Jurkat cells as the effectors, because these are well characterized T-cells and have high transfection efficiency. Clearly, it will be important to examine other effector cells, particularly primary human cells, with these vectors in the future. A variety of lymphoid and monocyte cell lines were examined as targets in cocultures with Jurkat cells transfected with HTLV-1 vectors. All cell lines were susceptible to infection, but Raji/CD4 cells gave the highest levels of Luc activity. This was not due to expression of CD4, as Raji cells gave similar levels of activity (data not shown). It is possible but unlikely that the CMV promoter-driven reporter gene is more active in Raji cells compared to other cell lines, even other EBV-transformed B-cell lines. Alternatively, higher levels of Luc transduction in Raji cells may reflect a unique interaction between adhesion molecules on the surface of Raji cells and Jurkat cells that enhances cell-to-cell infection. We are currently examining these possibilities. Although Raji/CD4 cells are not natural targets for HIV-1 infection, we believe that they do provide an appropriate model system to study HTLV-1 cell-to-cell infection, as B-cells are natural targets for HTLV-1 infection *in vivo* and *in vitro* [35],[36],[37],[38],[39] and can form synapses with HTLV-1-infected T lymphocytes *in vitro* [14]. While it is desirable to examine cell-to-cell infection using primary T-cells as targets, we have been unable to detect Luc transduction in cocultures of transfected Jurkat T-cells and activated CD4^+^ T-cells. This appears to be due to a negative effect of the activated CD4^+^ T-cells on VLP expression in Jurkat cells during the 48 h coculture infection, as the primary CD4^+^ T-cells (from various donors) were susceptible to infection with cell-free HIV-1 VLPs (D.M. and D.D., unpublished observation).

Infections carried out with HTLV-1 vectors in Jurkat-Raji/CD4 coculture had all of the characteristics expected for cell-to-cell infection. Infection was dependent on cytoskeletal remodeling, as inhibitors of actin and tubulin abolished infectivity. The time course of infection revealed rapid and efficient HTLV-1 VLP transmission immediately after mixing effector and target cells, and the difference in cell-free versus coculture infection was consistent with cell-to-cell infection for HTLV-1. Thus, we believe that this cell culture model system will be useful for examining the cell and virus determinants of cell-to-cell infection for HTLV-1. Relative to HTLV-1, HIV-1 VLPs appeared to be transmitted efficiently as cell-free particles. However, the inhibitory effect of the tubulin depolymerization agent nocodazole on HIV-1 coculture infection shows a cell-to-cell component of HIV transmission in our experimental setting. The differences in kinetics of HIV-1 coculture infection versus HTLV-1 infection may also be due to the less efficient or not too rapid VS formation between HIV producer cells and target cells. Thus, early after Raji/CD4 cell addition, cell free infection may predominate, but later transmission through the VS may be favored. The release of HIV from the surface of producer cells needed for viral maturation and recent demonstration of HIV endosomal fusion [47] may influence the rate of infectious transmission. HTLV-1 Gag is found to be processed inside the cells [40],[48],[49], so infectious VLPs can be readily fused with plasma membrane and quickly transfer the infection. HIV Jurkat-Raji/CD4 coculture infection displays a high resistance to neutralizing mAbs (25–50 µg/ml for 80–95% inhibition in kinetics experiments versus 5–10 µg/ml for cell-free infection (DM, DD unpublished observations), another indication of cell mediated virus transfer. This is consistent with previous reports of HIV cell-to-cell transmission being resistant to broadly neutralizing Abs and patient serum [10],[26],[50]. Therefore, to estimate cell-to-cell infection of HIV *per se*, the cell-free component of infection should be eliminated experimentally or subtracted from a total level of infectivity measured in cell cocultures.

We showed that the HTLV-1 Tax protein is a major contributor to the difference between HTLV-1 and HIV-1 infection in Jurkat-Raji/CD4 cocultures and that Tax significantly enhanced HTLV-1 cell-to-cell infection. The positive effects of Tax on HTLV-1 infection observed in the Jurkat-Raji/CD4 cocultures were not detected in 293T cells, indicating that Tax enhances infection in a cell type-specific manner. Tax is known to modulate many cellular functions, affecting the orientation and activity of the microtubule organizing center (MTOC) [51], up-regulating the expression of cell adhesion molecules such as ICAM-1 [52], and modulating signal transduction pathways [44]. Furthermore, Tax has been observed to localize to an area in infected T-cells near the interface with the target cell [51]. We suspect that multiple activities of Tax may cooperate to enhance cell-to-cell infection; examination of various Tax mutants, which are defective for specific interactions with cellular proteins, may help to identify critical Tax actions. The likelihood that Tax induces formation of a specialized cell adhesion synapse for efficient viral transmission is suggested by the result showing that a superantigen-induced immunological synapse between Jurkat cells and Raji/CD4 cells did not enhance cell-to-cell infection. Although IS has similarity with VS, the formation of the SEE-induced IS is inappropriate for VLP transmission. Recent reports demonstrating that HIV Gag preferentially forms ring [23] or wide “button” [26] like structures at VS, i.e. localizes in a peripheral, not central, supramolecular adhesion complex of synapse, further highlight the differences between IS and VS.

In summary, we have developed an experimental system, which makes it possible to directly quantify cell-to-cell infection. Results obtained with this system underscore the importance of quantitative measurements to validate inferences based on microscopic observations. It will be extremely interesting to extend the experimental approach described here to other cell types and, first of all, to primary human cells, and we are optimizing transfection and coculture conditions for such experiments.

## Materials and Methods

### Cells, antibodies, and reagents

Human cell lines Jurkat E6-1, JM, MOLT-4, SupT1, CEM174, H9, AA-2, Ramos, U937 and THP-1 were obtained through the AIDS Reference Reagent Program, Division of AIDS, NIAID, NIH. Raji/CD4 cells [31] were from Vineet N. KewalRamani (NCI-Frederick) and 729B cells were from Patrick Green (Ohio State University). T-cell, B-cell and monocyte cell lines were maintained in RPMI 1640 medium containing 10% fetal calf serum. Primary human CD4+ T cells were prepared from elutriated lymphocytes and grown in RPMI 1640 medium containing 10% fetal calf serum and 100 U per ml of IL-2 after activation with anti-CD28/anti-CD3 beads as described previously [53]. Human kidney 293T cells, Hela-P4 cells [54] (Eric Freed, NCI-Frederick), hybridomas anti-human CD4 clone SIM.2 and SIM.4, anti-HIV-1 gp120 clone 902 (NIH AIDS Research & Reference Reagent Program) were grown in Dulbecco's modified Eagle's medium (DMEM) containing 10% fetal calf serum. IgG from hybridoma supernatants were purified using protein A (for IgG1) or protein G (for IgG2b) HiTrap columns (GE Healthcare), then desaulted using PD-10 column (GE Healthcare), reconstituted in Dulbecco PBS without Ca and Mg and sterilized by filtration through 0.45 micron low protein-binding filters (Millipore). The anti-human CD3 clone UCHT1 (unconjugated, FITC-conjugated, or APC-conjugated) and anti-human HLA-DR clone TU36 and clone G46-6 (conjugated with PE) mAbs were from BD Pharmingen. Goat anti-mouse Alexa 546 secondary antibody was from Molecular Probes. Inactivated plasma from HTLV-1-positive patients was from Scripps Laboratories. Jasplakinolide was from Molecular Probes; cytochlasin D was from Calbiochem; azidothymidine and nocodozole were from Sigma. Staphylococcal enterotoxin E (SEE) was purchased from Toxin Technology (Sarasota, Florida).

### Plasmids

The HTLV-1 packaging plasmid pCMVHT1-M expresses the full-length HTLV-1 genome and pCMVHT1M-ΔEnv expresses all HTLV-1 gene products except Env [42]. The HTLV-1 packaging plasmids, pCMVHT1M-Tax9Q and pCMVHT1M-ΔEnv-Tax9Q are Tax-minus due to a single nucleotide change creating a stop codon in place of the glutamine at Tax codon 9. The HIV-1 packaging plasmid pCMVΔ8.2R expresses all HIV-1 proteins except Env [55]. pCMV-HT1Env and pCMV-VSVG express HTLV-1 Env and VSV-G protein, respectively. The HIV-1 Env expression plasmid pIIINL-4env [56] was obtained from Eric Freed (NCI-Frederick). The HTLV-1 Tax expression plasmid pCMV-Tax1C was described [57]. The dominant negative form of human VPS4A protein was expressed from pGFP-VPS4A-E223Q [58]. Lentivirus vectors for transduction of HTLV-1 Tax and GFP were constructed by subcloning HTLV-1 Tax-IRES-GFP or IRES-GFP cassettes into pUCHR transfer vector to give pUCHR-TaxIRGFP and pUCHR-IRGFP, respectively.

New, replication-dependent HTLV-1 reporter vectors were made from pHTC-GFPLuc [42] by first replacing the U3 region in the 5′LTR with CMV promoter, joined at the TATA box. The reporter cassette within the vector was replaced with a cassette from the plasmid pKS99gfp (from John Moran, University of Michigan), containing CMV promoter, *gfp* gene (with γ-globin intron), and TK polyA signal [29]; this cassette is oriented in the opposite direction relative to viral vector transcription, but the intron is oriented in the sense direction. Finally, the *gfp* gene was replaced with either *luciferase* or *yfp* genes containing a γ-globin intron to give pCRU5HT1-inLuc and pCRU5HT1-inYFP, respectively (which we refer to as HTLV1-inLuc and HTLV1-inYFP). HIV-1 replication-dependent reporter vectors, pUCHR-inLuc and pUCHR-inYFP were constructed from pUCHR-GFPLuc by replacing the reporter cassette with the respective cassettes from HTLV-1 vectors described above, and are referred to here as HIV1-inLuc and HIV1-inYFP.

### Transfections and infections

Jurkat cells, 293T cells and Hela-P4 cells were transfected with *Trans*IT®-Jurkat, *Trans*IT®-293 (Mirus), or FuGENE6 (Roche) transfection reagents, respectively, according to the manufacturers' instructions. Cell-free infection assays were performed essentially as described previously [59]. VLP concentrations were determined by HTLV-1 p19 or HIV-1 p24 antigen capture ELISA (Zeptometrix). Coculture infections were initiated by transfecting Jurkat cells (10^6^ cells in 1 ml) with 0.6 µg of inLuc vector DNA, 0.4 µg of packaging plasmid DNA, and 0.1 µg of Env-expression plasmid DNA (for env-minus packaging plasmids) or empty vector DNA (for env-positive packaging plasmids). After 24 h, cells were washed twice with PBS and 10^6^ Jurkat cells were mixed with an equal number of Raji/CD4 target cells in 5 ml of medium. Cells were collected 48 h later, extracted in GLO lysis buffer (Promega), and Luc activity was measured using Promega luciferase reagent and Lumat LB9501 luminometer (Berthold). For antibody blocking experiments, HTLV-1-positive human plasma was heated at 56°C for 30 min. to inactivate complement and added to HTLV-1 infections at 1/100 dilution; similarly treated HIV-1-positive human plasma was used as a control for HTLV-1 infections. For HIV-1 infections, anti-gp120 902 mAb at a concentration 50 µg/ml or anti-CD4 SIM.2 mAb 25 µg/ml was added to cells; non-blocking anti-CD4 SIM.4 mAb at a similar concentration was used as control. Immunological synapse formation was induced by pulsing Raji/CD4 cells (10^7^ cells per ml in PBS) with 10 µg/ml of SEE for 1 hr; cells were washed 4 times with PBS, and combined with Jurkat cells at a 1∶1 ratio.

### FACS analysis of cell conjugate formation

Untreated or SEE-treated Raji/CD4 cells (10^7^ per ml) were mixed with an equal number of Jurkat cells in complete medium for 1 hr. After centrifugation, cells were fixed with 4% paraformaldehyde for 10 min, washed with PBS, stained with anti-CD3-FITC plus anti-HLA-DR-PE Abs for 30 min, and analyzed by flow cytometry. Cell conjugates were expressed as the percentage of double positive cells in the total Jurkat cell population. To determine the effects of HTLV-1 Tax expression on adhesion of Jurkat cells to Raji/CD4 cells, Jurkat cells were transduced with cell-free VLPs that were generated by transfecting 293T cells with pUCHR-TaxIRGFP or pUCHR-IRGFP plus pCMVΔ8.2R and pCMV-VSVG. At 48 hrs after transduction, Jurkat cells (greater than 85% GFP-positive) were washed and mixed with Raji/CD4 cells at a 1∶1 ratio for 1 hr. Cell conjugate formation was determined as described above.

## Supporting Information

Figure S1 Morphological and phenotypic analysis of infected cells using inYFP vectors. (A) FACS analysis of infection events. Jurkat cells were transfected with inYFP vectors and respective packaging plasmids and cocultured with Raji/CD4 cells in the presence or absence of 20 µm AZT. YFP-positive cells were enumerated by flow cytometry 48 hr after infection; dead cells were excluded by propidium iodide (PI) staining. (B) Aliquots of cells were removed from the samples described above 48 hr after infection and placed on glass coverslips. Cells were fixed and stained with anti-HLA-DR antibody to mark Raji/CD4 cells (red), with DAPI to stain nuclei (blue), or with anti-CD3 antibody to stain Jurkat cells (not shown). Cells were analyzed by fluorescent deconvolution microscopy; typical fields are presented as optical sections through the middle of cells. Arrows indicate infected, YFP-positive cells (green). (C) YFP-positive cells from two slides for each virus were counted and categorized according to cell type and morphology. YFP-positive cells were generally mononuclear; i.e. single Jurkat cells or single Raji/CD4 cells. Rarely, a YFP-positive Raji/CD4 cell was observed in the process of fusion with a large multinucleated syncytium (fusing Raji) or a small region of a giant HLA-DR-positive cell displayed YFP fluorescence (giant fused cells DR^+^).
(1.13 MB TIF)

Figure S2 Time course of HIV-1 entry block by antibodies to Env or CD4. Mouse monoclonal antibodies, produced in hybridomas 902 (anti-gp120) and SIM.2 (anti-hCD4) were purified and concentrated, as described in method section, then titrated to give at least 85% inhibition of HIV-1 infection with 902 mAb and greater than 95% inhibition with SIM.2 mAb. To evaluate the kinetics of infection, at the times indicated in the figure mAbs were added to cocultures of Jurkat and Raji/CD4 cells (where 0 hr marks the time when cells were mixed). Luciferase activity was then measured 48 hr after the start of coculture. The mean of three independent experiments with standard deviations shown with error bars are presented. As demonstrated on the figure, either Ab against the Env (filled squares) or CD4 receptor (open squares) efficiently and similarly inhibited HIV-1 replication in a slow rate manner. Neither SIM.2 nor 960 mAbs inhibited HTLV-1 cell-to-cell infection (data are not shown), confirming the specificity of blocking.
(0.05 MB TIF)

Figure S3 Coculture infectivity of HIV-1 and HTLV-1 VLPs are affected differently by Env pseudotyping. Jurkat cells transfected with inLuc reporter vector, Env-minus packaging plasmid, and variable amounts of the indicated Env-expression plasmids were cocultured with Raji/CD4 cells. Infectivity was measured by luciferase assay 48 hr after infection. (A) HTLV-1 VLPs were pseudotyped with HTLV-1 Env (squares), HIV-1 Env (triangles), or VSV-G protein (circles). (B) HIV-1 VLPs were pseudotyped with HTLV-1 Env (squares), HIV-1 Env (triangles), or VSV-G protein (circles). Infectivity is expressed as luciferase activity (RLU) normalized to the amount of Gag protein in the supernatant at the end of infection. The data represent the mean of at least of three independent experiments with error bars indicating standard deviation.
(0.12 MB TIF)

Protocol S1 FACS analysis and immunofluorescent microscopy of infected cells.
(0.03 MB DOC)
