# Supplementary figures and images for: Quantitative Comparison of HTLV-1 and HIV-1 Cell-to-Cell Infection with New Replication Dependent Vectors
Source: PLoS Pathog. 2010 Feb 26;6(2):e1000788. doi: 10.1371/journal.ppat.1000788 (PMC2829072; doi:10.1371/journal.ppat.1000788)

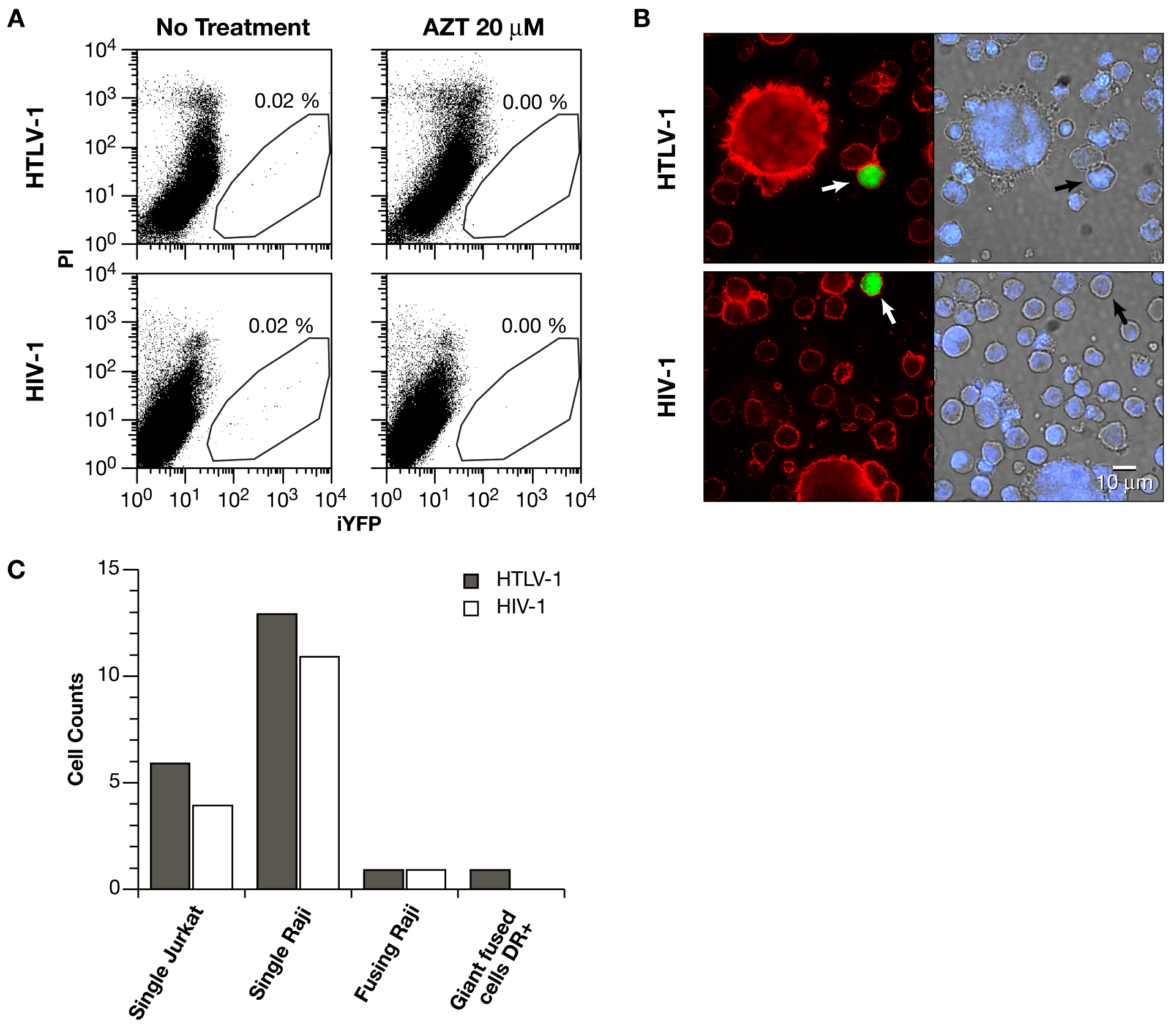

Supplement: Figure S1 — Morphological and phenotypic analysis of infected cells using inYFP vectors. (A) FACS analysis of infection events. Jurkat cells were transfected with inYFP vectors and respective packaging plasmids and cocultured with Raji/CD4 cells in the presence or absence of 20 µm AZT. YFP-positive cells were enumerated by flow cytometry 48 hr after infection; dead cells were excluded by propidium iodide (PI) staining. (B) Aliquots of cells were removed from the samples described above 48 hr after infection and placed on glass coverslips. Cells were fixed and stained with anti-HLA-DR antibody to mark Raji/CD4 cells (red), with DAPI to stain nuclei (blue), or with anti-CD3 antibody to stain Jurkat cells (not shown). Cells were analyzed by fluorescent deconvolution microscopy; typical fields are presented as optical sections through the middle of cells. Arrows indicate infected, YFP-positive cells (green). (C) YFP-positive cells from two slides for each virus were counted and categorized according to cell type and morphology. YFP-positive cells were generally mononuclear; i.e. single Jurkat cells or single Raji/CD4 cells. Rarely, a YFP-positive Raji/CD4 cell was observed in the process of fusion with a large multinucleated syncytium (fusing Raji) or a small region of a giant HLA-DR-positive cell displayed YFP fluorescence (giant fused cells DR+). (1.13 MB TIF) [file ppat.1000788.s001.tif]

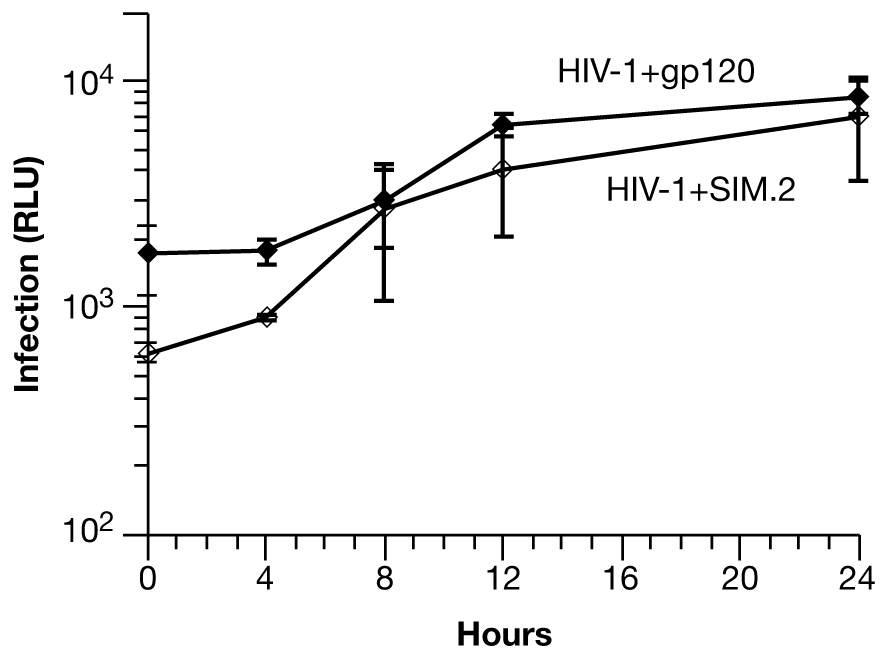

Supplement: Figure S2 — Time course of HIV-1 entry block by antibodies to Env or CD4. Mouse monoclonal antibodies, produced in hybridomas 902 (anti-gp120) and SIM.2 (anti-hCD4) were purified and concentrated, as described in method section, then titrated to give at least 85% inhibition of HIV-1 infection with 902 mAb and greater than 95% inhibition with SIM.2 mAb. To evaluate the kinetics of infection, at the times indicated in the figure mAbs were added to cocultures of Jurkat and Raji/CD4 cells (where 0 hr marks the time when cells were mixed). Luciferase activity was then measured 48 hr after the start of coculture. The mean of three independent experiments with standard deviations shown with error bars are presented. As demonstrated on the figure, either Ab against the Env (filled squares) or CD4 receptor (open squares) efficiently and similarly inhibited HIV-1 replication in a slow rate manner. Neither SIM.2 nor 960 mAbs inhibited HTLV-1 cell-to-cell infection (data are not shown), confirming the specificity of blocking. (0.05 MB TIF) [file ppat.1000788.s002.tif]

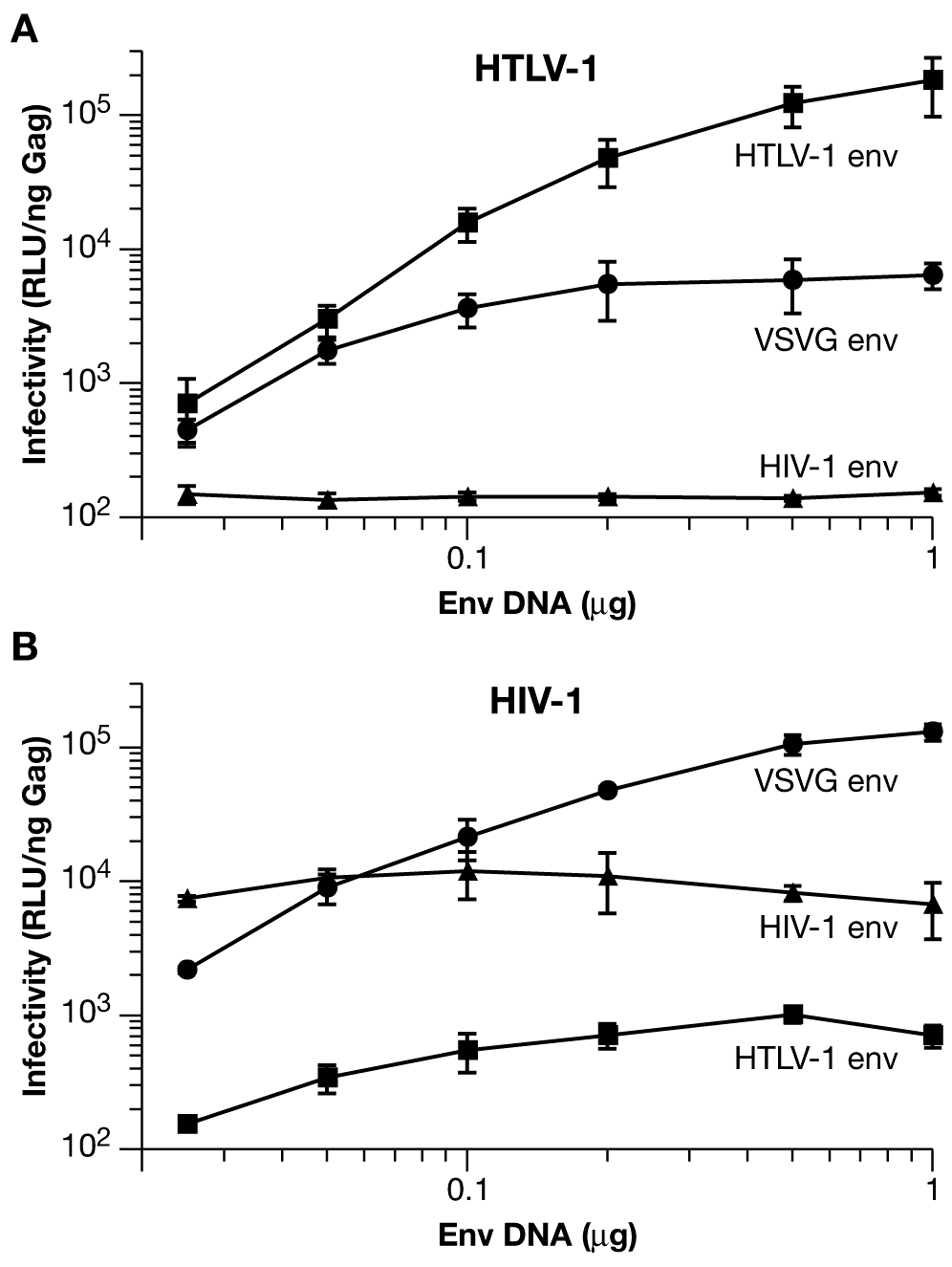

Supplement: Figure S3 — Coculture infectivity of HIV-1 and HTLV-1 VLPs are affected differently by Env pseudotyping. Jurkat cells transfected with inLuc reporter vector, Env-minus packaging plasmid, and variable amounts of the indicated Env-expression plasmids were cocultured with Raji/CD4 cells. Infectivity was measured by luciferase assay 48 hr after infection. (A) HTLV-1 VLPs were pseudotyped with HTLV-1 Env (squares), HIV-1 Env (triangles), or VSV-G protein (circles). (B) HIV-1 VLPs were pseudotyped with HTLV-1 Env (squares), HIV-1 Env (triangles), or VSV-G protein (circles). Infectivity is expressed as luciferase activity (RLU) normalized to the amount of Gag protein in the supernatant at the end of infection. The data represent the mean of at least of three independent experiments with error bars indicating standard deviation. (0.12 MB TIF) [file ppat.1000788.s003.tif]
