# Supplementary material for: Quantitative Comparison of HTLV-1 and HIV-1 Cell-to-Cell Infection with New Replication Dependent Vectors
Source: PLoS Pathog. 2010 Feb 26;6(2):e1000788. doi: 10.1371/journal.ppat.1000788 (PMC2829072; doi:10.1371/journal.ppat.1000788)
Supplement: Protocol S1 — FACS analysis and immunofluorescent microscopy of infected cells. (0.03 MB DOC) [file ppat.1000788.s004.doc]

FACS analysis and immunofluorescent microscopy of infected cells. For HTLV-1, Jurkat cells (2x10^6^ cells) were transfected with 3μg of HT1-inYFP transfer vector and 2 μg of pCMVHT1-M packaging plasmid. For HIV-1, Jurkat cells (2x10^6^ cells) were transfected with 3μg of HIV1-inYFP transfer vector, 2 μg of pCMVΔ8.2R packaging plasmid, 1.5 μg of pIIINL-4env, and 2.5 μg of pCMV-Tax1C. Transfected Jurkat cells were co-cultured with an equal number of Raji/CD4+ cells with or without 20 μm AZT. To increase YFP expression levels, cell cultures were stimulated with 10 ng per ml of PMA 16 hrs prior to luciferase assay. The percentage of infected cells was evaluated by flow cytometry (FACSCalibur, BD) in the presence of 2 μg/ml propidium iodide (PI); 2x10^5^ events were plotted in FL-1 (inYFP) versus FL-2 (PI) coordinates. To visualize the infection events, cells from samples without AZT treatment were adhered to poly-L-lysine-coated glass coverslips in PBS for 30 min, fixed with 4% paraformaldehyde (Sigma) for 20 min, washed, and stained with mouse anti-CD3 or anti-HLA-DR antibody for 30 min. Secondary goat anti-mouse Alexa 546 antibody was added to washed cells in PBS in the presence of 1% normal goat serum for another 30 min. After washing, coverslips were stained with DAPI solution, transferred to slides, and maintained in the ProLong antifade Kit (Invitrogen, Molecular Probes). Slides were analyzed on a Delta Vision RT deconvolution microscope (Applied Precision, LLC). Image data were analyzed with the SoftWoRx Imaging Work station.
